# P-959. Antimicrobial Stewardship Team’s Relationship with Enterobacterales Bloodstream Infection: It's Complicated

**DOI:** 10.1093/ofid/ofaf695.1161

**Published:** 2026-01-11

**Authors:** Kazumi Morita, Jacqueline Burnell, Daniel Mueller

**Affiliations:** Temple University Hospital, Philadelphia, Pennsylvania; Temple University, Philadelphia, Pennsylvania; Temple University Hospital, Philadelphia, Pennsylvania

## Abstract

**Background:**

Shorter antibiotic durations have shown similar clinical outcomes to longer durations for the treatment of uncomplicated Enterobacterales bloodstream infection (uE-BSI). As an antibiotic stewardship (AS) initiative, we developed an institution-approved guideline focused on identification of uE-BSI, early oral antibiotic transition, and shorter antibiotic duration with a process for prospective audit and feedback (PAF). The purpose of this study was to evaluate the incidence of uE-BSI and describe the impact of PAF at a quaternary medical center.

**Methods:**

A real-time alert triggered by the electronic medical record (EMR) including patients with positive blood cultures for Enterobacterales was reviewed on weekdays by the AS team during 4/1/2024-9/18/2024. uE-BSI was defined as meeting all the following criteria: Known bacteremia etiology, achievement of source control, no metastatic foci, clinical improvement on appropriate antibiotic therapy for 72 hours, and immunocompetence. Patients with polymicrobial organisms mixed with non-Enterobacterales, and with situations where diagnostic work up was incomplete (e.g. death or patient directed discharge) were considered as complicated infection.

**Results:**

The AS team reviewed the EMR alerts for 155 patients with Enterobacterales bacteremia and 30 patients (19.4%) met criteria for uE-BSI with urinary source being the most common (70%). Among patients with uE-BSI, the AS team intervened on 6 (3.9% of all 155 patients) patients and missed 13 (8.4%) AS opportunities due to patient discharge prior to PAF. Median duration of antibiotic therapy for uE-BSI was shorter in the 17 patients who had an infectious disease (ID) consult or AS team intervention than those 13 patients who did not (8d vs. 14d, p=0.04)

**Conclusion:**

At our institution, low prevalence of uE-BSI was observed and PAF did not yield robust AS interventions. However, as shorter antibiotic durations for uE-BSI were selected more frequently with ID or AS team involvement, AS initiatives to optimize the treatment of uE-BSI are beneficial. As PAF is a labor-intensive process, exploring innovative processes by leveraging the EMR to streamline the identification of patients with uE-BSI may be a useful adjunct to PAF that could increase the impact of this initiative.

**Disclosures:**

All Authors: No reported disclosures

